# Semi-automatic Extraction of Plants Morphological Characters from Taxonomic Descriptions Written in Spanish

**DOI:** 10.3897/BDJ.6.e21282

**Published:** 2018-06-26

**Authors:** Maria Auxiliadora Mora, José Enrique Araya

**Affiliations:** 1 Costa Rica Institute of Technology (ITCR), Cartago, Costa Rica

**Keywords:** Information Extraction, Natural Language Processing, Biodiversity Informatics

## Abstract

Taxonomic literature keeps records of the planet's biodiversity and gives access to the knowledge needed for its sustainable management. Unfortunately, most of the taxonomic information is available in scientific publications in text format. The amount of publications generated is very large; therefore, to process it in order to obtain high structured texts would be complex and very expensive. Approaches like citizen science may help the process by selecting whole fragments of texts dealing with morphological descriptions; but a deeper analysis, compatible with accepted ontologies, will require specialised tools. The Biodiversity Heritage Library (BHL) estimates that there are more than 120 million pages published in over 5.4 million books since 1469, plus about 800,000 monographs and 40,000 journal titles (12,500 of these are current titles).

It is necessary to develop standards and software tools to extract, integrate and publish this information into existing free and open access repositories of biodiversity knowledge to support science, education and biodiversity conservation.

This document presents an algorithm based on computational linguistics techniques to extract structured information from morphological descriptions of plants written in Spanish. The developed algorithm is based on the work of Dr. Hong Cui from the University of Arizona; it uses semantic analysis, ontologies and a repository of knowledge acquired from the same descriptions. The algorithm was applied to the books Trees of Costa Rica Volume III (TCRv3), Trees of Costa Rica Volume IV (TCRv4) and to a subset of descriptions of the Manual of Plants of Costa Rica (MPCR) with very competitive results (more than 92.5% of average performance). The system receives the morphological descriptions in tabular format and generates XML documents. The XML schema allows documenting structures, characters and relations between characters and structures. Each extracted object is associated with attributes like name, value, modifiers, restrictions, ontology term id, amongst other attributes.

The implemented tool is free software. It was developed using Java and integrates existing technology as FreeLing, the Plant Ontology (PO), the Plant Glossary, the Ontology Term Organizer (OTO) and the Flora Mesoamericana English-Spanish Glossary.

## Introduction

The transformation of texts from taxonomic literature into structured data remains a key challenge in biodiversity informatics, recognised by international initiatives such as the Global Biodiversity Information Facility (GBIF), the Encyclopedia of Life (EOL), and the Biodiversity Heritage Library (BHL) (Hobern et al. 2013, Thessen and Parr 2014, Salle et al. 2009). The BHL estimates that there are more than 120 million pages published in over 5.4 million books since 1469, plus about 800,000 monographs and 40,000 journal titles (12,500 of these are current titles) (Rinaldo et al. 2009). It is necessary to develop data standards and software tools to extract, integrate and publish this knowledge into existing free and open access repositories to support science, education and biodiversity conservation.

Taxonomic literature keeps records of the planet's biodiversity and gives access to the knowledge needed for its sustainable management. The scientific community has described more than 1.9 million species, which represents around 17% of the planet's expected biodiversity (Chapman 2009). The taxonomic work, expressed in a simplified way, consists of organising all forms of life ideally in a hierarchy, assigning a Latin name to each taxon, a taxonomic category that associates it to a level in the hierarchy, a morphological description, a diagnostic description that is sometimes accompanied by diagnostic drawings, habitat description, information about its distribution, and identification keys, amongst other information.

Morphological descriptions synthesise observations made by taxonomists over centuries of research. They contain statements that detail morphological aspects (i.e. shape and structure) of species useful to identify it. Statements may describe structures, substructures, characters, states, and relationships between structures. Examples of structures are leaves, apex, flowers, or fruits. Examples of characters are length, width, pigmentation patterns, smell, or architecture. An example of part of a description is the statement "hojas simples, alternas, 4-10 (-14) × 1-3.3 cm. / Simple leaves, alternate, 4-10 (-14) × 1-3.3 cm.". In this example, the described structures are the "hojas / leaves", one of their characters is the architecture (not mentioned) and the state of this character is "simple". To take full advantage of this information and to work towards integrating it with repositories of biodiversity knowledge such as the ones developed by GBIF, EOL and BHL, the biodiversity informatics community first needs to convert plain text into a machine-processable format. More precisely, it is needed to identify structures and substructures names and the characters that describe them. The generated data would allow, amongst other services, the development of applications to identify specimens (e.g. electronic keys), to improve information search mechanisms, to perform data analysis of species having particular characteristics, and to compare species descriptions.

**Figure 1.**
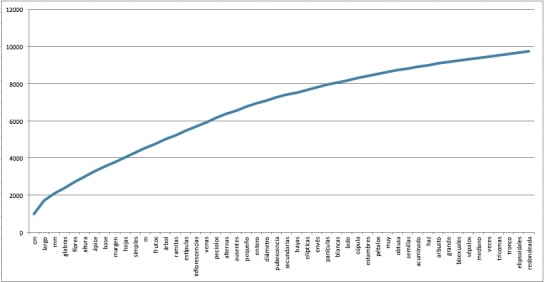
Cumulative frequency of the fifty most used words included in the book Trees of Costa Rica volume IV; they represent about 30% of the total words. Only adjectives, adverbs and names were considered.

Fortunately, morphological descriptions of plants use a semi-structured language characterised by:

- Employing many abbreviations and omitting functional words and verbs, making sentences to become telegraph-like phrases to save space in scientific publications and field guides;
- Texts are written in a very technical language because the formal terminology is based on Latin;
- The characters, in most cases, are not explicit, for example the phrase "Flores blancas / White flowers" does not mention the "colour" character. In order to assign a character name, ontologies and controlled vocabularies are used;
- They contain mostly names, adjectives, numbers (measures) and, to a lesser extent, adverbs. Verbs are used very infrequently.
- The vocabulary used is repetitive. Fig. 1 shows a cumulative frequency graph of the fifty most used words in the morphological descriptions of the book TCRv4 considering only names, adjectives, and adverbs. These represent about 30% of all words used in them.
- They use a highly standardised syntax even though they are written in natural language. Table 1 gives examples of types of phrases used in TCRv4 book descriptions.

Biodiversity Informatics (BI) provides techniques and mechanisms to capture, process, integrate, and publish data and information on the planet's biodiversity. BI initiatives, such as GBIF, EOL, BHL, and the Bar Code of Life, work on the discovery, aggregation and free exchange of genetic data, species occurrences, natural history information, conservation status, management, conservation and geographic data, amongst others. The integrated data enable users to answer questions related to processes that occur in time and space, for example, the possible effects of climate change on particular species, the effects of land-use change on species in an area, prediction of the introduction routes of invasive species, amongst others. To the aforementioned data types, species traits databases have been added more recently. They store in the form of triplets (e.g. flowers, colour, white) information extracted manually or semi-automatically from morphological descriptions, habitat, natural history, species interactions, and distribution. An example of such repositories is the TraitBank designed by EOL to integrate data from multiple databases (Parr et al. 2016).

This research is related to the information extraction area (Hobbs and Riloff 2010) and aims to structure semi-automatically morphological characters of plant species written in Spanish, using semantic analysis techniques, ontologies, and a knowledge repository acquired from the contents of the same descriptions. While there has been a significant amount of research performed over the last few years on extracting information from taxonomic literature, a few of them have been orientated to structure the complete content of the species' morphological description and no documented effort has been directed toward structuring information written in Spanish (Thessen et al. 2012).

The implemented system will define the basis to continue the work of processing more than one hundred field guides of plant and other biological groups such as arthropods, mollusks, vertebrates, and fungi published by the National Biodiversity Institute of Costa Rica (INBio) and, in the future, to support the Latin American community in this process. To process this information manually constitutes a monumental task and algorithms like the one proposed in this research would greatly alleviate the large effort required.

The following are the major contributions of this research:

- It presents a semi-automatic algorithm to extract plant morphological characters from Spanish text.
- This is a novel contribution because work on this area for Spanish is very rare or non-existent.
- The base approach taken from Dr. Cui's work was adjusted to deal with the particularities that processing Spanish content adds to the complexity of the process and to the evaluation of the results.
- Very good results were obtained in many aspects of the annotation for morphological descriptions: annotation of structures, annotation of characters, associate characters with the appropiate structures and processing of conjunctions.
- It will provide a ground base for future research like preposition processing in Spanish.

## Material and methods

With the present investigation, an effort has been made to generate structured information semi-automatically with high semantic value from morphological descriptions of plants written in Spanish. The goal of the algorithm is to extract structures, substructures, characters states, to relate each character to its corresponding structure or substructure, and to establish relationships between structures. Specifically, the algorithm must convert morphological descriptions, that are initially in text format, into records of a database.

**Figure 2.**
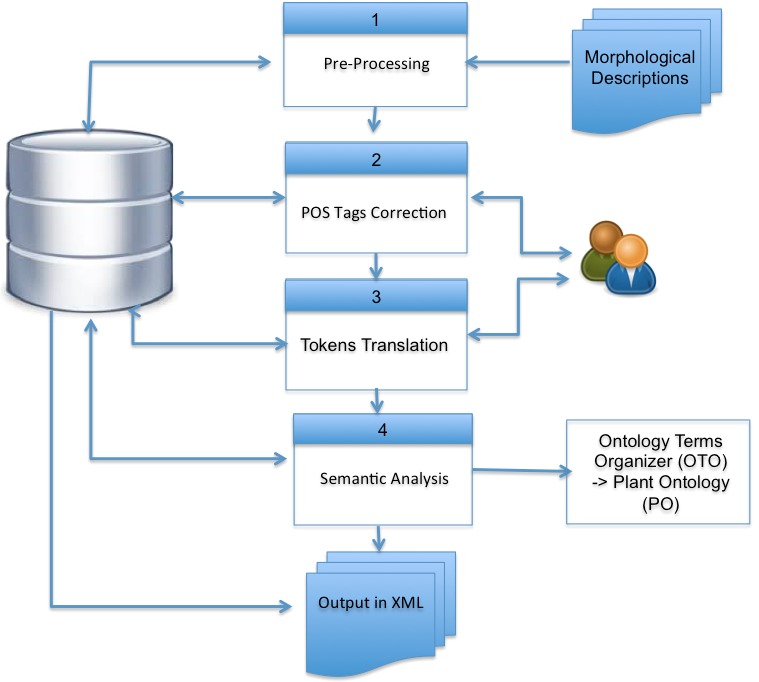
Flowchart of the implemented algorithm.

The system receives morphological descriptions in tabular format (i.e. scientific name and description), processes them, and generates documents in XML according to the scheme proposed by Cui (2012). The schema permit the documentation of structures, characters, constraints, and relationships between structures and characters. The system processes taxonomic descriptions by means of the following steps (Fig. 2 shows the system data flow):

1. *Pre-processing*: Descriptions are standardised by removing double and single quotes, replacing abbreviations, and other transformations. They are segmented into clauses using end point, colons, and semicolons as separators. Clauses are sentences that, in case of plant species, completely describe a structure. Additionally, each clause is segmented into chunks using commas as separators. Chunks are the atomic units of processing.
2. *Part-of-Speech (POS) Tags Correction* (Hippisley 2010): The FreeLing's Morphosyntactic Analyzer (Padró and Stanilovsky 2012) assigns a POS tag to each word according to the role it plays in the sentence (e.g. name, adjective, adverb, determinant). Due to the semi-structured language used in morphological descriptions and to the technical terminology based on Latin, FreeLing correctly assigns POS tags only to adverbs, determinants, pronouns, conjunctions, prepositions, numerals, and dates. POS tags assigned to verbs, names and adjectives must be evaluated and corrected in some cases. Performing this process manually is a time-consuming task, therefore bootstrapping, an unsupervised machine learning algorithm based on rules, was implemented (McDermott and Charniak 1985). The algorithm implements an iterative and incremental learning process in which knowledge is deduced from an existing knowledge base. In each cycle, new knowledge about the role that tokens play in a chunk is integrated into the knowledge base. For instance, a rule could be defined as: for two or more tokens separated by the preposition "a", if at least one of them is labelled as a character state (type=A), then the others will also be labelled as a character state. For the chunk “redondeada a cordada a subcordada / rounded to chordate to subcordate“, "rounded" was initially labelled as character state then the algorithm was also labelled "chordate" and "subcordate" as a character state (Jones et al. 1999).
3. *Tokens Translation*: Ontologies are a very valuable and limited resource in the field of BI and amongst those that are available, not many of them manage Spanish translations of terms. The PO (Plant Ontology Consortium 2002, Avraham et al. 2008) includes Spanish translations in the form of synonyms, however, in this research, it was decided to use OTO because of the great advantage it has of being a collaborative effort that allows specialists to organise and share their terms. OTO currently integrates some ontologies, including the PO (Huang et al. 2015). Nevertheless, as OTO does not integrate synonyms, consequently to match the states of characters with those included in OTO, it was necessary to translate terms into English using first Google Translator (with a 82.5% success in translating terms of the morphological descriptions of ACRv4) and then integrating the Flora Mesoamericana English-Spanish Glossary (a web-based interface developed by the Missouri Botanical Garden as part of the Flora Mesoamericana publication, Fernando et al. 2017).
4. *Semantic Analysis*: A complete description is available in section **Semantic Annotation of Descriptions**.

**Figure 3.**
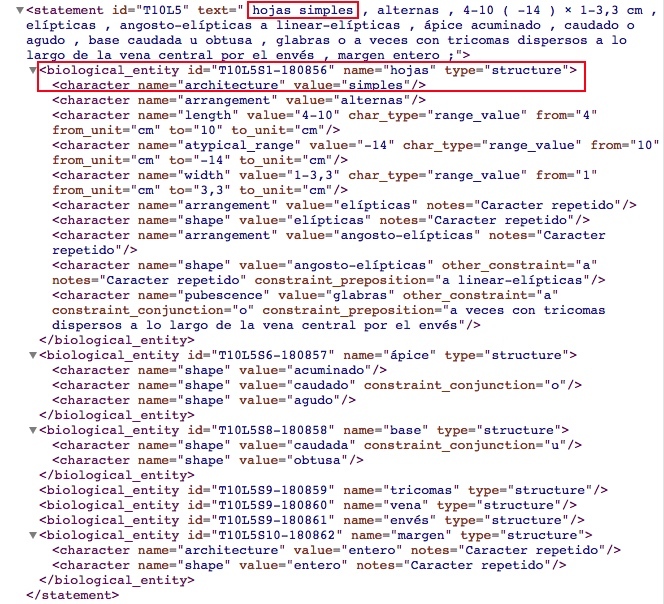
Example of how the system structured a clause part of the description of the species *Quercus salicifolia* from the book TCRv4.

Fig. 3 shows an example of how the system structured the following clause of the book TCRv4: Original clause: "hojas simples, alternas, 4-10 (-14) × 1-3.3 cm., elípticas, ápice acuminado, caudado o agudo, base caudada u obtusa, glabras o a veces con tricomas dispersos a lo largo de la vena central por el envés." English translation: "Simple leaves, alternate, 4-10 (-14) × 1-3.3 cm, elliptic, apex acuminate, caudate or acute, base caudate or obtuse, glabrous or sometimes with trichomes dispersed along the central vein on the underside."

As can be seen in the red frame on Fig. 3, the phrase "hojas simples / simple leaves" generated a structure for the "hojas / leaves" and a character for the "architecture" with "simples / simple" state. The name of each character (e.g. "architecture") is obtained from the Plant Glossary (Endara et al. 2017) because these elements commonly do not explicitly appear in the description. The Plant Glossary is used through OTO, given a state and OTO web services returns the characters that include this state in its controlled vocabulary. The characters that fulfil this condition could be more than one, in the example of the Fig. 3, the "elliptical" state is associated with "arrangement" and "shape" as can be observed in the following lines:

- <character name="arrangement" value="elípticas" notes="Carácter repetido"/>
- <character name="shape" value="elípticas" notes="Carácter repetido"/>

The system adds a note of "Carácter repetido / Repeated character" so that, at a later stage, an expert could determine the correct character to be used.

### Semantic annotation of descriptions

The rules and conditions that guide the extraction process were defined from a morphosyntactic analysis performed on descriptions of the TCRv4 book (Ljunglof and Wirén 2010). Rules were derived when processing the most common types of grammatical structures used by authors, for instance name + adjective (e.g. "flores rojas / red flowers") used in more than 17.5% of chunks of the book and chunks with only one adjective (e.g. "deciduous / deciduous") used more than 12.5%.

The algorithm analyses the role of each token and the dependencies between tokens in a chunk and creates or modifies the corresponding objects in a database. Three types of objects are generated: structures, characters, and relationships. Tokens that do not generate one of those types of objects are considered modifiers. Chunks, that are part of the same clause, are processed from left to right. The order of processing is important because not all chunks include the structure with which the characters should be associated and therefore the algorithm should look for it in previously processed chunks.

**Figure 4.**
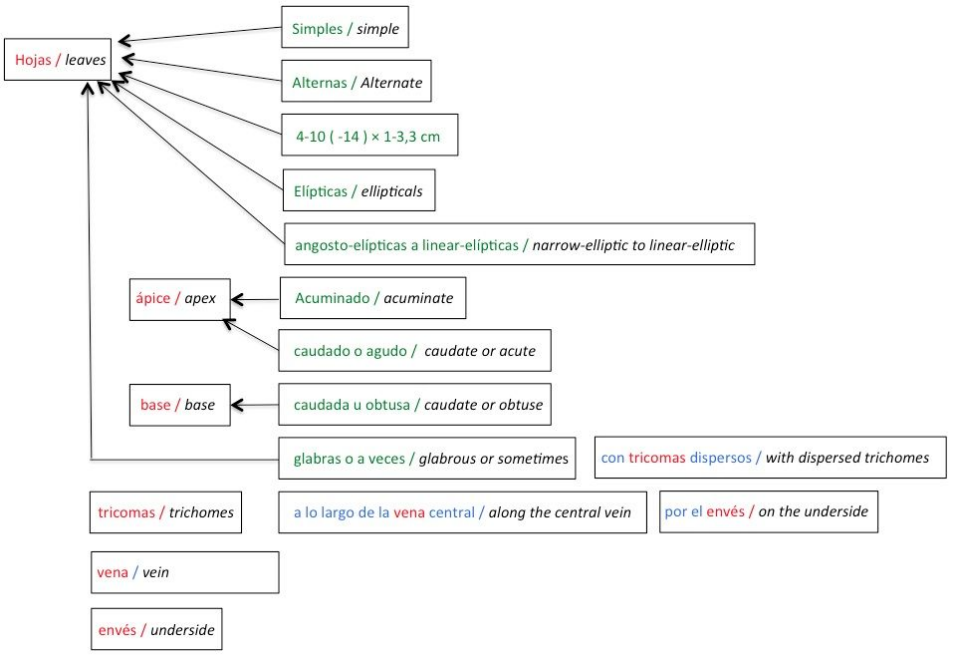
Example of matching process between tokens using simple rules based on gender and number for *Quercus salicifolia* (book TCRv4).

To associate each character with the correct structure or substructure, the algorithm uses the order of appearance of tokens and their concordance in gender and number. Fig. 4 shows structures (written in red), the characters states (in green) and the relationship between structures and characters (as an arrow). In this example, the "simple", "alternate", "elliptic", and "narrow-elliptic to linear-elliptic" characters must be associated with the "leaves" structure; "acuminate" and "caudate or acute" to the "apex" substructure; and "caudate or obtuse" to the "base" structure. Nevertheless, "glabrous" must be associated with "leaves" because it does not match gender and number with the closest substructures (i.e. base). The structures "trichomes", "vein", and "underside" are part of prepositional phrases and the system does not process all the information in them; however, structures are extracted for subsequent use of them during the association process.

**Figure 5.**
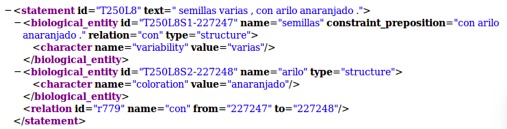
Example of how the system structured a prepositional phrase that starts with the token "con / with" (clause T250L8).

The algorithm does not extract all information available in taxon descriptions. It does not process all prepositional or verbal phrases, however, as a proof of concept, the prepositional phrases that begin with tokens "sin / without" or "con / with" are structured. The rest of prepositional or verbal phrases are only delimited as constraint_preposition and constraint_verb respectively. Fig. 5 shows an example of how the system structured a phrase that begin with "con / with". The XML section includes a relationship named "con / with" from the structure "semillas / seeds" (227247) to the structure "arilo / aryl" (2272248).

**Figure 6.**
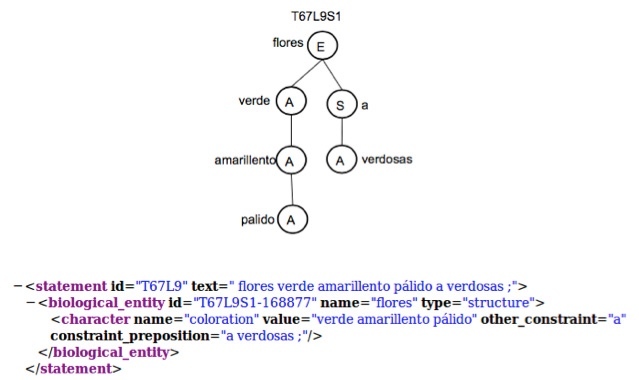
Dependency tree and structured text for chunk "flores verde amarillento pálido a verdosas / flowers pale yellowish-green to greenish".

Semantic annotation is done using dependency trees generated by the FreeLing Dependency Parser (Padró and Stanilovsky 2012), knowledge acquired by the system and a set of rules associated with the position of tokens in the tree (i.e. its role inside a chunk) and its neighbouring nodes (i.e. ancestor, siblings, and children). Some of the defined rules or conditions are listed below:

**Adjectives (A) rules**:

- Adjectives are represented by an A in a dependency tree. Nodes containing an adjective instantiate an object of the Character Class.
- Two or more adjectives that are related (i.e. ancestor or child) must be included in the same object when they complement their meaning. Two adjectives complement their meaning if they have the same character name. Example "flores verde amarillento pálido a verdosas / flowers pale yellowish-green to greenish". In this case the character name for all adjectives is "colouration". The dependency tree and the structured text are shown in Fig. 6.

**Names (E) rules**:

- Names or entities are represented by an E in a dependency tree. Nodes containing an entity or a name instantiate an object of the Structure Class.
- Two related names (i.e. ancestor or child) must be part of the same object. The second structure is a modifier of the first one. Example "frutos nueces / nut fruits".

**Figure 7.**
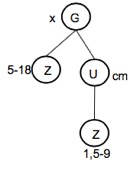
Dependency tree of the chunk "5-18 x 1.5-9 cm".

**Figure 8.**
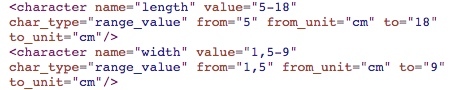
Result of structuring the chunk "5-18 x 1.5-9 cm".

**Figure 9.**
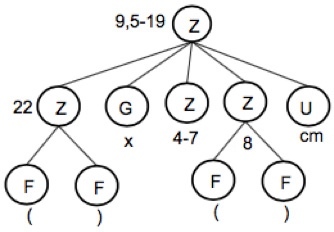
Dependency tree of chunk "9.5-19 (-22) × 4-7 (-8) cm"

**Figure 10.**
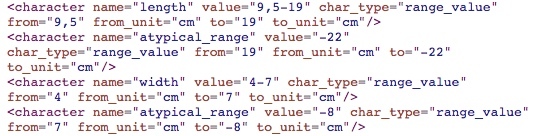
Result of structuring the chunk "9.5-19 (-22) × 4-7 (-8) cm"

**Numerals (Z) rules**:

- Numerals are represented by a Z in a dependency tree. They define quantities, sizes or areas. Numerals instantiate an object of the Character Class.
- Numeric ranges describe areas. In Botany, the first part of the range corresponds to the object's length and the second part to the object's width. For numeric ranges, the system fills the "from" and "to" properties of the character. Example, "5-18 x 1.5-9 cm". In this example two characters are created, one for the length (5-18) and the other for the width (1.5-9). The first one has the attributes name = "length", value = "5-18", char_type = "range_value", from = "5", from_unit = "cm", to = "18", to_unit = cm". The dependency tree and the structuring results are shown in Fig. 7 and Fig. 8.
- Atypical range values are presented in parentheses before or after a numeral. For instance, in "9.5-19 (-22) × 4-7 (-8) cm", (-22) defines an atypical range value ranging from 19 to 22 cm (19 not included). Atypical ranges may occur before or after a number and must be processed depending on whether they are at the left or right side of this number. Fig. 9 and Fig. 10 present the dependency tree and results of structuring the previous example.

**Figure 11.**
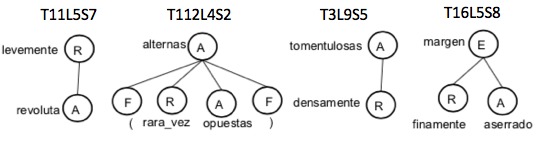
Dependency trees for examples of use of adverbs in Table 2.

**Figure 12.**
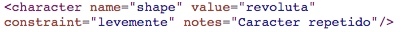
Structured text that exemplifies the use of adverbs in chunk T11L5S7.

**Figure 13.**
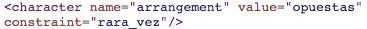
Structured text that exemplifies the use of adverbs in the chunk T112L4S2.

**Figure 14.**
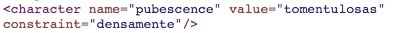
Structured text that exemplifies the use of adverbs in the chunk T3L9S5.

**Figure 15.**
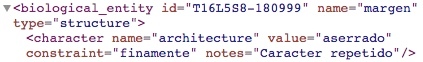
Structured text that exemplifies the use of adverbs in the chunk T16L5S8.

**Figure 16.**
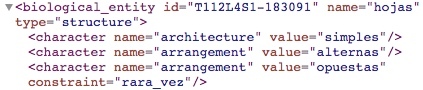
Structured text that exemplifies the use of adverbs in the chunk T112L4S2.

**Adverbs (R) rules**:

- Adverbs are represented by an R in a dependency tree. They modify the structure or character with which they have a dependency relationship. Even though they mostly modify characters, sometimes in complex chunks, they can modify structures. Table 2 shows some examples of the use of adverbs. The dependency trees for these examples are shown in Fig. 11.
- Adverbs are stored in the constraint attribute of the structure or character to which they modify.
- The association to one or another type of object (Structure or Character) obeys the following rules:
  - If adverb R1 has a child C1 that is an adjective, verb, number, unit of measure, structure or adverb, then R1 is associated C1. Example T11L5S7. The structured text is shown in Fig. 12.
  - If adverb R1 has a sibling B1 that is an adjective, verb, number, unit measure, structure or adverb, then R1 is associated to B1. Example T112L4S2. The structured text is shown in Fig. 13.
  - If adverb R1 has a parent P1 that is an adjective, verb, number, unit measure, structure, or adverb, then R1 is associated to this P1. Example T3L9S5. The structured text is shown in Fig. 14.
- An example of associating an adverb with a character is shown in Fig. 15. The chunk T16L5S8 has the text "finely serrated margin". In this case, the character "aserrado / sawn" and the constraint = "finamente / finely" associated with it are created.
- If adverb R1 appears in a sentence in parentheses, the algorithm associates R1 with the token that corresponds within the sentence as shown in Fig. 16. Example chunk T112L4S2 "alternas (rara vez opuestas) / alternating <leaves> (rarely opposed)".

**Figure 17.**
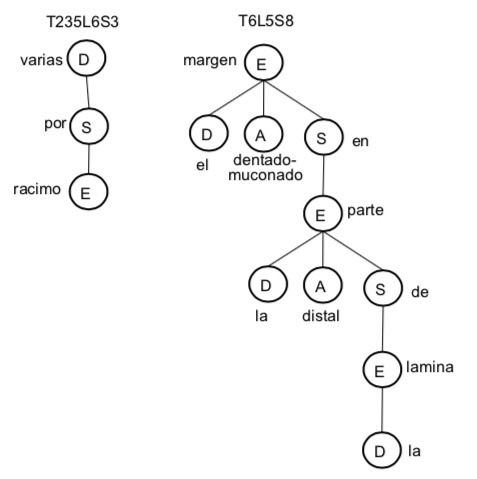
Dependency trees for examples that use determiners in Table 3.

**Figure 18.**
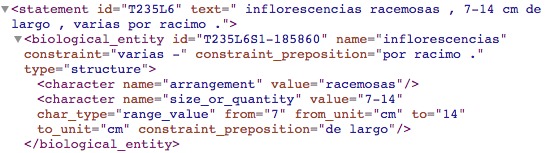
Structured text that exemplifies the use of determiners in the chunk T235L6S1.

**Determiners (D) rules**:

The algorithm processes quantifiers (i.e. some, quite, none, all, several) and articles (i.e. the) as a proof of concept. Table 3 presents some examples of chunks using determiners and the dependency trees are shown in Fig. 17. Quantifiers are applied to structure as modifiers. The structured text of example T235L6S3 is shown in Fig. 18.

Other elements such as articles, pronouns, and conjunctions are processed in the same way by the algorithm.

## Data resources

The texts used in this research are from the books Trees of Costa Rica Volume III - ACRv3 (Zamora et al. 2004) and Trees of Costa Rica Volume IV - ACRv4 (Zamora et al. 2011) provided by the lead author of both publications, Nelson Zamora and a subset of descriptions of the Manual of Plants of Costa Rica - MPCR (Grayum et al. 2003) provided by Dr. José Enrique Araya with the authorisation of Nelson Zamora (one of the authors of the MPCR). The descriptions of the MPCR were semi-automatically extracted as part of a research project for extraction of knowledge from biological literature led by Dr. Araya. That project was sponsored by the Costa Rica Institute of Technology (ITCR). ACRv4 was used for the development of the algorithm. ACRv3 and MPCR were used for the algorithm evaluation.

## Results

In this section, we describe the results of executing the algorithm in a random sample of clauses extracted from the ACRv3 and MPCR books (5% of the total available clauses). The data sample was produced using the Roulette Wheel selection algorithm (Sivanandam and Deepa 2008), which allows the assignment of more priority to clauses with more structures (as a complexity indicator). Table 4 presents the average number of structures and characters per clause for each book.

The ACRv3 book includes information on 233 species with 1,738 clauses, of which 87 (5%) were included in the data sample. From the MPCR, 237 species descriptions were selected from which a random sample of 106 (5%) was extracted.

**Figure 19.**
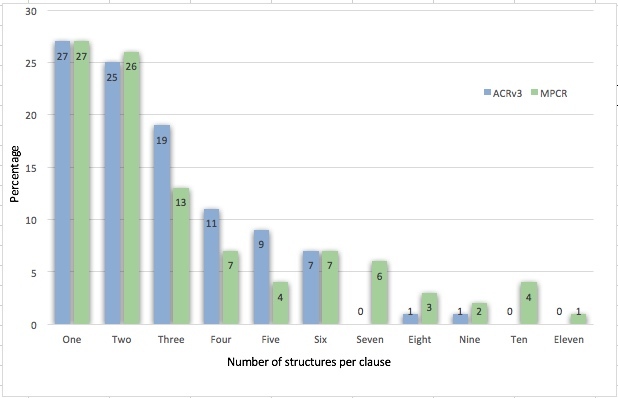
Complexity (number of structures) per clause in the samples of the ACRv3 and MPCR books.

The complexity of the clauses in the samples taken from the ACRv3 and MPCR books were well distributed. 52% of clauses were simple and 48% complex in ACRv3 and 53% of clauses were simple and 47% complex in MPCR. It is estimated that a clause is simple if it has two or less structures and complex with more than two structures. Fig. 19 shows the number of structures per clause in the sample of the ACRv3 and MPCR books.

The metrics generally used in IE to evaluate the results are precision and recall (Hobbs and Riloff 2010). They measure the percentage of correct annotations and the completeness of the extraction method, respectively. In addition, the F-1 (the harmonic mean between precision and coverage) was used. The precision, recall, and F-1 were calculated for each individual sample of each book (Tables 5, 6, 7 show the results).

The following datasets used by this research are available in GitHub (Mora and Araya 2017):

- an XML file with the fragments with the morphological descriptions.
- a zip file that contains XML files with all species morphological descriptions structured after processing.
- a PDF file showing the evaluation of each of the cases randomly selected.

That site also contains a detailed description of the files.

## Discussion

The semantic annotation results showed that, due to the semi-structured nature of morphological descriptions of plants, it is feasible to implement, with excellent results, a simple semantic analysis algorithm based on rules using available technology (i.e. FreeLing, OTO, PO, and Flora Mesoamericana English-Spanish Glossary). From Table 7, it can be seen that good results can be achieved by the algorithm with more than 92.5% of average performance in annotating structures, characters, associating characters with structures, and processing conjunctions.

The algorithm is scalable (within the biological group of plants) as demonstrated by evaluating it not only in tree records (ACRv3), but also in records of aquatic plants, shrubs, epiphytes, grasses, and lianas described by different authors of the MPCR. Although the MPCR clauses are somewhat more complex as shown in Fig. 19,the degradation of the algorithm was acceptable. Performance went from 97.9% on average when evaluating book ACRv3 to 92.5% when evaluating MPCR. The greatest drawback when processing the MPCR book was the association of characters with structures (F = 86.4), which shows that the simple heuristic of character / structure association by gender and number agreement must be complemented with the use of ontologies or other resources (e.g. an expanded knowledge base).

**Figure 20.**
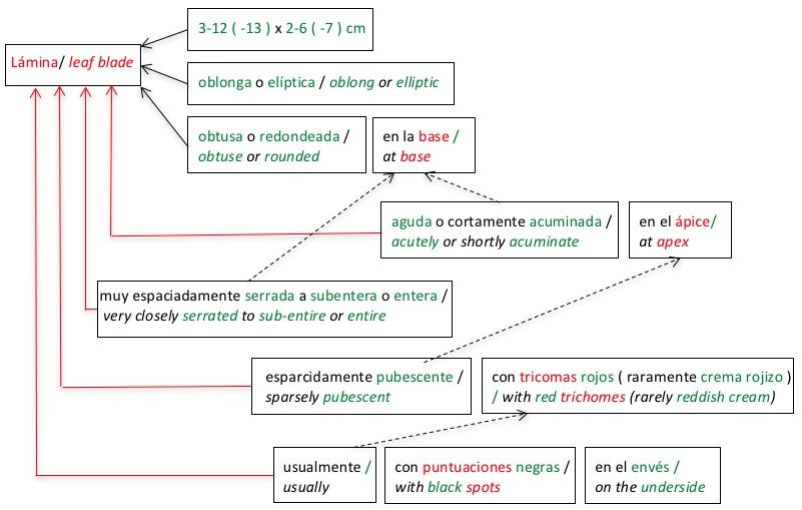
Diagram showing the error in assigning characters to structures using the simple gender and number agreement heuristic in clause T520L3 describing the species *Hydrangea asterolasia* (MPCR).

Fig. 20 exemplifies the problem of associating characters with structures for the description of the shrub *Hydrangea asterolasia* (included in the MPCR). Original clause: "lámina 3-12 (-13) x 2-6 (-7) cm, oblonga o elíptica, obtusa o redondeada en la base, aguda o cortamente acuminada en el ápice, muy espaciadamente serrada a subentera o entera, esparcidamente pubescente con tricomas rojos (raramente crema rojizo), usualmente con puntuaciones negras en el envés."

English translation: "Leaf blade 3-12 (-13) x 2-6 (-7) cm, oblong or elliptic, obtuse or rounded at base, acutely or shortly acuminate at apex, very closely serrated to sub-entire or entire, sparsely pubescent with red trichomes (rarely reddish-cream), usually with black spots on the underside."

In this example, all chunks should be associated with the main structure (Fig. 20 shows a solid red line if an association error exists). However, they were associated with substructures following the heuristic of gender and number agreement with the previously processed structure (dotted line). For example "aguda o cortamente acuminada en el ápice / *acute or short acuminate at the apex*" was associated with "base" instead of being associated with "lámina / *leaf blade*".

The performance of the algorithm can be improved using ontologies which include hierarchies of structures / substructures and controlled vocabularies (list of valid characters to describe a structure) or storing additional information in the knowledge base, as follows:

1. Evaluate the chunk context to determine the structure with which the characters should be associated. For instance, in Fig. 20, the characters ("shape", "acutely or shortly acuminate") were associated with the "base" structure and, if the chunk context had been evaluated in "acute or shortly acuminate at the apex", it would have been evident that the "apex" was not part of the "base" but of the "leaf blade". This conclusion can be reached by using hierarchies of structures / substructures (managed by ontologies or included in the knowledge base).
2. Before associating a character with a structure, the algorithm must evaluate whether the association is correct. For example, the "serrated" architecture is not an option to describe the base of the leaves, it is rather an option to describe the "leaf blade". This conclusion can be reached using controlled vocabularies.

This result should be considered if the algorithm is going to be extended for application to other biological groups (i.e. vertebrates or arthropods) since there are no ontologies for all of them.

**Prepositional and Verbal Phrases**: The algorithm does not process all prepositional or verbal phrases, however, as a proof of concept, the prepositional phrases that begin with tokens "without" or "with" were structured. The rest of the prepositional or verbal phrases were only delimited as constraint_preposition and constraint_verb, respectively. With the results of this investigation and in a later refinement of the algorithm, the scope of the extraction goal in these cases should be defined in more detail. The refinement should take into account the meaning of each preposition and verb to process the chunk.

Fig. 5 shows the result of processing clause T250L8 "semillas varias, con arilo anaranjado / *various seeds, with orange aryl*". In this example, the algorithm created a relationship named "with" from the structures "seeds" to the structure "aryl".

**Figure 21.**
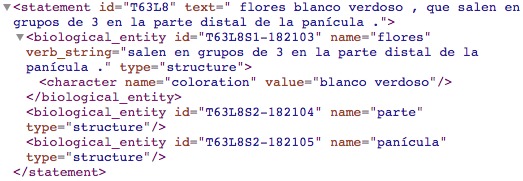
Example of structuring a verbal phrase (clause T63L8).

Fig. 21 presents an example of how the algorithm process verbs (clause: "white-green flowers, which come out in groups of 3 in the distal part of the panicle"). In this example, the algorithm processes the verb "come out" by delimiting the rest of the sentence with the verb_string tag.

This research presents an algorithm based on computational linguistics techniques to extract structured information from morphological descriptions of plants written in Spanish. It achieves very competitive results (more than 92.5% of average performance) in annotating structures, characters, associating characters with structures and processing conjunctions.

The algorithm is based on rules defined after analysing the morphosyntactic patterns of the dependency trees for the most-used grammatical structures in the ACRv4 book. To define those rules, 73.72% of the book's chunks were analysed.

As the good results of the system depend strongly on the fact that the roles assigned to the words in a chunk are correct, therefore it was necessary to implement a machine learning algorithm to correct the POS labels assigned by the FreeLing's Morphosyntactic Analyzer. The technical terminology based on Latin and the semi-structured language used in morphological descriptions, which is full of names, adjectives and adverbs with few verbs, make FreeLing misassign POS tags to names, adjectives, and verbs.

Although the algorithm achieved a very good performance, it is important to make improvements in some of the stages of the process that are listed below:

- Translation of tokens into English: This is the manual process that requires more user attention. Possible improvements include the use of synonyms of the PO; addition of other glossaries; use of Google synonyms; and incorporation of Wiktionary which, in most definitions, includes the botanical meaning of each term.
- Semantic annotation of descriptions: Part of the information available in morphological description was not extracted because it was part of a prepositional or verbal phrase. The scope of the information extraction process should be extended in order to structure the information contained in these phrases. Additionally, the selection of the appropriate character amongst the repeated characters could be done using machine learning algorithms.

The implemented algorithm is based on the telegraphic language used by the community of botanical experts. However, it can be generalised to other biological groups by preprocessing the texts of the descriptions to omit some functional words (e.g. the verb "to be") that bring them closer to the telegraphic language used by botanists and extending the functionality of the algorithm.

Ontologies are an important resource to extract information from morphological descriptions since they include functionality to agree on the characters that describe a structure / substructure, to document hierarchies of structures and substructures, and to define the controlled vocabulary for improving the association of characters to structures. However, not all biological groups have general ontologies such as the PO. An ontology integrator such as OTO or a knowledge base designed to include this information will help to improve results and to further develop the algorithm to work with other biological groups.

## Figures and Tables

**Table 1.** Examples of types of phrases (chunks) used in morphological descriptions included in TCRv4 (total number of phrases = 2,457). File is semi-colon separated.

**Phrase**	**Grammar structure (Spanish)**	**Amount of phrases with this structure**	% **of occurrence in text**
inflorescencias fasciculadas. / fasciculated inflorescences.	Name + adjective + punctuation mark (NAF)	1152	17.5
deciduas, / deciduous,	Adjective + punctuation mark (AF)	828	12.5
6-30 m de altura. / 6-30 m high.	number + unit of measurement + preposition + name + punctuation mark (ZUSNF)	613	9.8
6-30 × 2-10.5 cm	Number + area symbol +number + name +punctuation mark (ZGZNF)	289	4.2

**Table 2.** Examples of use of adverbs in the book ACRv4.

**Chunk**	**Contents**
T11L5S7	levemente revoluta / slightly revolute
T112L4S2	hojas simples, alternas (rara vez opuestas) / simple leaves, alternating (rarely opposed)
T3L9S5	densamente tomentulosas / densely tomentose
T16L5S8	margen finamente aserrado / finely sawed margin

**Table 3.** Examples of chunks that include determiners.

**Chunk**	**Contents**
T235L6S1	varias por racimo / *several per cluster*
T6L5S8	el margen dentado-mucronado en la parte distal de la lámina / *dentate-mucronate margin in the distal part of the lamina*

**Table 4.** Average number of structures and characters in the evaluated clauses of the ACRv3 and MPCR books.

**Book**	**Number of Descriptions**	**Total of Clauses**	**Sample Size** **(Clauses)**	**Average of**	
				**Structures**	**Characters**
ACRv3	233	1,738	87 (5%)	2.85	3.62
MPCR	237	2,230	106 (5%)	3.42	3.69

**Table 5.** Precision of the algorithm when applied to samples of ACRv3 and MPCR books.

**Book**	**Identification of structures (precision)**	**Character structuring (precision)**	**Association of characters to structures (precision)**	**Association of conjunctions (precision)**
ACRv3	97.9%	98.1%	98.7%	96.4%
MPCR	98.1%	92.8%	86.4%	92.4%

**Table 6.** Recall of the algorithm when applied to samples of ACRv3 and MPCR books.

**Book**	**Identification of Structures** (Recall)	**Character Structuring (Recall)**	**Association of characters to structures** **(Recall)**	**Association of conjunctions** **(Recall)**
ACRv3	97.9%	99.0%	98.7%	96.4%
MPCR	98.1%	93.0%	86.4%	92.4%

**Table 7.** Performance (F) of the algorithm when applied to samples of the ACRv3 and MPCR books.

**Book**	**Identification of Structures (F-1)**	**Character Structuring (F-1)**	**Association of characters to structures** **(F-1)**	**Association of conjunctions** **(F-1)**	**Average (F-1)**
ACRv3	97.9	98.5	98.7	96.4	97.9
MPCR	98.1	93.3	86.4	92.4	92.5
